# Description of Osmolyte Pathways in Maturing *Mdx* Mice Reveals Altered Levels of Taurine and Sodium/Myo-Inositol Co-Transporters

**DOI:** 10.3390/ijms23063251

**Published:** 2022-03-17

**Authors:** Caroline Merckx, Gwenny Cosemans, Jana Zschüntzsch, Robrecht Raedt, Jens Schmidt, Boel De Paepe, Jan L. De Bleecker

**Affiliations:** 1 Department of Neurology, Ghent University and Ghent University Hospital, 9000 Ghent, Belgium; caroline.merckx@ugent.be (C.M.); gwenny.cosemans@ugent.be (G.C.); jan.debleecker@ugent.be (J.L.D.B.); 2 Department of Neurology, University Medical Center Göttingen, 37075 Göttingen, Germany; j.zschuentzsch@med.uni-goettingen.de (J.Z.); j.schmidt@gmx.org (J.S.); 3 4BRAIN, Institute for Neuroscience, Department Head and Skin, Ghent University, 9000 Ghent, Belgium; robrecht.raedt@ugent.be; 4 Department of Neurology and Pain Treatment, Immanuel Klinik Rüdersdorf, University Hospital of the Brandenburg Medical School Theodor Fontane, 15562 Rüdersdorf bei Berlin, Germany; 5 Faculty of Health Sciences Brandenburg, Brandenburg Medical School Theodor Fontane, 15562 Rüdersdorf bei Berlin, Germany

**Keywords:** Duchenne muscular dystrophy, *mdx*, osmoregulation, taurine transporter, sodium myo-inositol transporter, aldose reductase

## Abstract

Duchenne muscular dystrophy (DMD) is a genetic disorder characterized by progressive muscle degeneration. Osmotic stress participates to DMD pathology and altered levels of osmolyte pathway members have been reported. The goal of this study was to gain insight in osmoregulatory changes in the *mdx* mouse model by examining the expression of osmolyte pathway members, including taurine transporter (TauT), sodium myo-inositol co-transporter (SMIT), betaine GABA transporter (BGT), and aldose reductase (AR) in the skeletal muscles and diaphragm of *mdx* mice aged 4, 8, 12, and 26 weeks. Necrosis was most prominent in 12 week-old *mdx* mice, whereas the amount of regenerated fibers increased until week 26 in the tibialis anterior. TauT protein levels were downregulated in the tibialis anterior and gastrocnemius of 4 to 12 week-old *mdx* mice, but not in 26 week-old mice, whereas TauT levels in the diaphragm remained significantly lower in 26 week-old *mdx* mice. In contrast, SMIT protein levels were significantly higher in the muscles of *mdx* mice when compared to controls. Our study revealed differential regulation of osmolyte pathway members in *mdx* muscle, which points to their complex involvement in DMD pathogenesis going beyond general osmotic stress responses. These results highlight the potential of osmolyte pathway members as a research interest and future therapeutic target in dystrophinopathy.

## 1. Introduction

Duchenne muscular dystrophy (DMD) is an X-linked muscle wasting disorder that affects approximately 1 in 5000 live male births [1]. Patients suffer from progressive muscle weakness and become wheelchair bound in their early teens. DMD is caused by a mutation in the dystrophin gene, resulting in loss of functional dystrophin protein. Dystrophin is a key component of the dystrophin-associated protein complex that plays an important role in the stabilization of the sarcolemma by connecting the intracellular cytoskeleton to the extracellular matrix. Dystrophin deficiency compromises the sarcolemmal integrity of myofibers and fibers are more susceptible to contraction-induced damage, leading to repetitive cycles of muscle degeneration and regeneration, inflammation, and fibrosis [2,3,4].

In muscle samples from DMD patients, edema and excessive sodium levels are present [5], suggesting osmotic perturbations play a role in DMD pathology. When cells are subjected to osmotic stress, they import or synthetize osmolytes to counteract the osmotic imbalance. This build-up of organic osmolytes is orchestrated by nuclear factor of activated T cells 5 (NFAT-5)-induced activity of genes coding for members of osmolyte pathways. The latter include aldose reductase (AR), an enzyme that catalyzes the reduction of glucose to sorbitol, and osmolyte transporters such as taurine transporter (TauT), betaine GABA transporter 1 (BGT), and the sodium/myo-inositol transporter 1 (SMIT), responsible for cellular import of osmolytes, respectively taurine, betaine, and myo-inositol. Osmolytes can also contribute to membrane stabilization and serve as anti-oxidants [3]. In muscle samples from DMD patients, increased protein levels of AR, TauT, and SMIT have been observed [6], which might support the regenerative processes taking place in dystrophin-deficient tissue.

The most widely used animal model for DMD is the *mdx* (X-chromosome-linked muscular dystrophy) mouse. Similar to DMD pathology in humans, the lack of dystrophin in the *mdx* mouse model manifests as loss of muscle force, inflammation, and myofiber necrosis. While the diaphragm of *mdx* mice shows progressive and severe deterioration over the whole life span, hindlimb muscle damage appears early in life and stabilizes afterwards. Approximately 3 to 4 weeks after birth, necrosis is reported in 30–60% of the myofibers in the tibialis anterior [7,8]. This period of extensive muscle damage is followed by repetitive cycles of degeneration and regeneration, after which necrosis persists further at lower levels. Within 7 to 10 days after necrosis, myoblasts become activated, proliferation and differentiation take place, and myoblasts fuse into myotubes, which mature over time [8,9,10]. Regenerated myofibers are identified by the presence of central nuclei, which appear around 1 to 4 days after replication of myoblasts and migrate from the center to the periphery in approximately 50–100 days [7,10,11]. Myofibers with one or more central nuclei thus represent the regenerative response to myofiber necrosis. Hence, the severity and progressive nature of the human disease is not fully reflected by the condition of skeletal muscles of the *mdx* mouse. Nonetheless, the unique features of this model allow the detailed assessment of changes in expression of osmolyte pathway members in relation to the age-dependent degenerative and regenerative processes occurring in the skeletal muscle of the *mdx* mouse.

Expression of TauT has been studied in the *mdx* mouse model, showing its significant downregulation in the quadriceps of *mdx* mice [12]. Our current study is, however, the first to investigate multiple osmoregulatory components in maturing *mdx* mice. The nature of osmo-deregulation in DMD remains poorly understood, therefore we aimed to further study the relation between osmoregulation and dystrophinopathology. We examined the expression of osmolyte pathway members using qPCR, immunofluorescence, and Western blot in the skeletal muscles and diaphragm of *mdx* mice at different ages, revealing differential expression patterns.

## 2. Results

### 2.1. Histopathological Characterization of the Maturing Mdx Mouse

**Figure 1:**
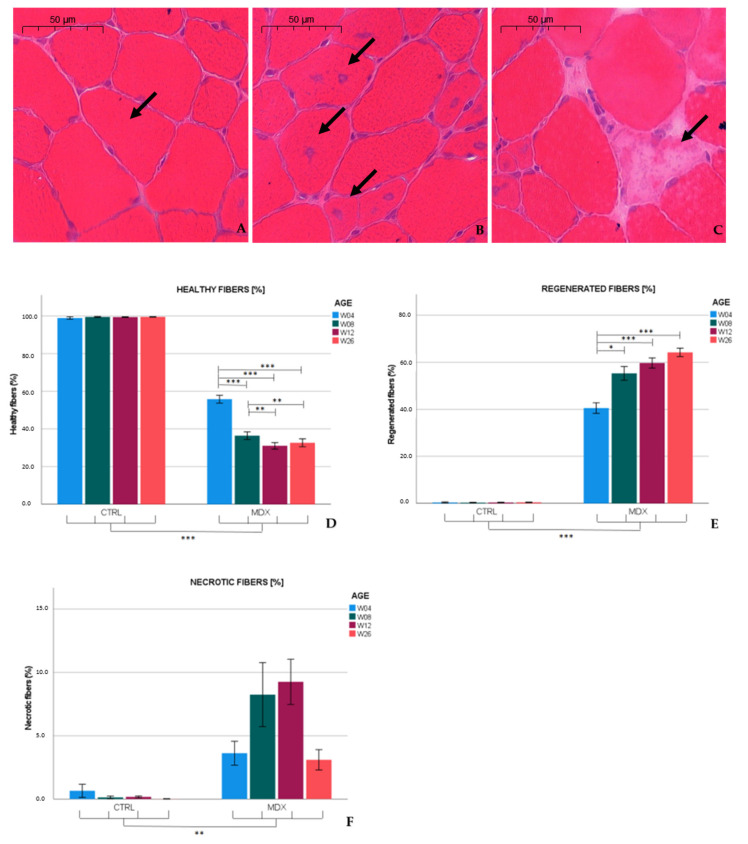
Histopathological characteristics of the *mdx* mouse model. (**A**–**C**) Haematoxylin-Eosin (H&E) stained *mdx* muscle sections. (**A**) Arrow indicates a healthy muscle fiber with nuclei at the peripheral site. (**B**) Arrows indicate a group of regenerating fibers with centralized nuclei. (**C**) Arrow indicates a necrotic fiber characterized by loss of membrane integrity and pale cytoplasmic aspect. (**D**) Quantification of the percentage of healthy fibers, (**E**) regenerated fibers with central nuclei, and (**F**) necrotic fibers counted in the tibialis anterior of *mdx* and control mice aged 4, 8, 12, and 26 weeks. Results were based on manual scoring of 5 H&E stained sections per animal taken approximately 150 µm apart (*n* = 5 animals per age group). A total of 199 sections were analyzed. Data presented as the mean ± standard error of the mean (SEM). Significant differences are annotated by * (*p* ≤ 0.05), ** (*p* ≤ 0.01) and *** (*p* ≤ 0.001).

Dystrophinopathy was assessed in Hematoxylin-Eosin (H&E) sections of the tibialis anterior derived from 4-, 8-, 12-, and 26-week-old *mdx* and age-matched control mice, and results are summarized in Figure 1. In every age group, *mdx* mice displayed significantly altered numbers of healthy (*p* ≤ 0.001), regenerated (*p* ≤ 0.001), and necrotic fibers (*p* ≤ 0.01) when compared to age-matched control animals. In 4 week-old *mdx* mice, about 40% of the myofibers had centralized nuclei, indicating previous necrotic damage. Necrosis gradually decreased by age 26 weeks, but the differences observed in young and older *mdx* mice were not statistically significant. In addition, the percentage of non-muscle area was quantified manually and through semi-automatic detection (Supplementary Figure S1). As expected, muscle damage was significantly higher in *mdx* mice when compared to control mice.

### 2.2. Protein Levels of Osmolyte Pathway Members in Skeletal Muscle and Diaphragm of the Mdx Mouse Model on Different Time Points

**Figure 2:**
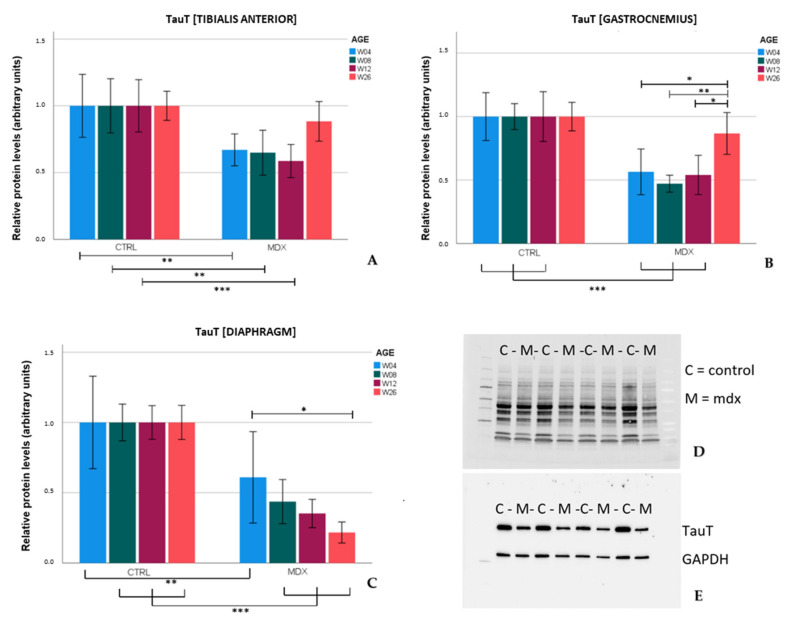
Relative protein levels of the taurine transporter (TauT) in skeletal muscle and diaphragm of *mdx* and control mice aged 4, 8, 12, and 26 weeks. Relative protein levels of TauT in tibialis anterior (**A**), gastrocnemius (**B**), and diaphragm (**C**). Representative image of total protein detected in the diaphragm of 12 week-old *mdx* and control mice by Stain-Free technology that was used to correct for variations in loading (**D**) and the corresponding image of TauT and glyceraldehyde 3-phosphate dehydrogenase (GAPDH) protein (**E**). Data are reported relative to protein levels in control mice within the same age category and shown as mean ± SEM and *n* = 7–8. Significant differences are annotated by * (*p* ≤ 0.05), ** (*p* ≤ 0.01) and *** (*p* ≤ 0.001).

Protein levels of TauT, as revealed by Western blot, in the tibialis anterior and gastrocnemius, were significantly lower in *mdx* mice in comparison to age-matched control mice at age 4, 8, and 12 weeks (*p* ≤ 0.01). At age 26 weeks, TauT levels almost returned to the levels observed in age-matched control mice. Interestingly, a different trend was observed in the diaphragm of *mdx* mice. TauT protein levels in *mdx* mice were significantly lower (*p* ≤ 0.01) in comparison to age-matched control mice and tended to further decrease with age (Figure 2). Similar trends were obtained when normalized against the housekeeping protein glyceraldehyde 3-phosphate dehydrogenase (GAPDH) (Supplementary Figure S2).

**Figure 3:**
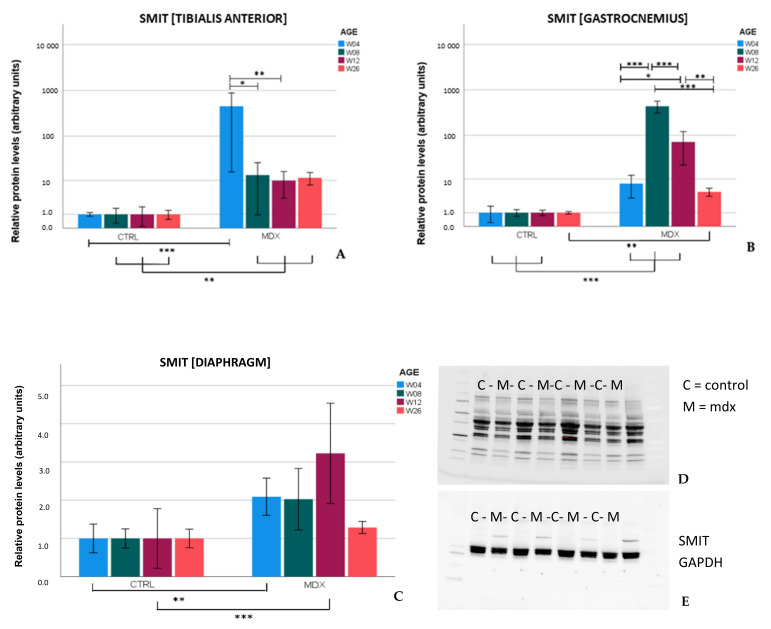
Relative protein levels of the sodium/myo-inositol transporter (SMIT) in skeletal muscle and diaphragm of *mdx* and control mice aged 4, 8, 12, and 26 weeks. Relative SMIT levels in tibialis anterior (**A**), gastrocnemius (**B**), and diaphragm (**C**). Representative image of total protein detected in the gastrocnemius of 12 week-old *mdx* and control mice by Stain-Free technology that was used to correct for variation in loading (**D**) and the corresponding image of SMIT and GAPDH protein (**E**). SMIT levels in *mdx* mice were reported relative to protein levels of SMIT in control mice within the same age category (*n* = 7–8). To adhere to normality assumptions, data underwent a log transformation. Log transformed data was used for statistics. Data is shown as mean ± SEM. Significant differences are annotated by * (*p* ≤ 0.05), ** (*p* ≤ 0.01) and *** (*p* ≤ 0.001).

Protein levels of SMIT in skeletal muscle of *mdx* mice were significantly (*p* ≤ 0.01) higher than in age-matched control mice at every examined time point, whereas in the diaphragm, significant differences were observed at ages 4 (*p* = 0.006) and 12 weeks (*p* ≤ 0.001) (Figure 3). In skeletal muscle of control mice, SMIT levels were very low, whilst in *mdx* mice, the SLC5A3 band could easily be detected in all samples. On the contrary, in the diaphragm, clear bands were present in both *mdx* and control mice, explaining the more moderate fold changes in diaphragm. The protein fold change between control and *mdx* mice is shown in Table 1. Additional normalization against housekeeping protein was performed (Supplementary Figure S3).

Table 1 gives an overview of the protein fold change in *mdx* mice relative to age-matched control mice in different muscles.

No changes were observed in AR levels between *mdx* and control mice at any age using stain-free blot normalization (Supplementary Figure S4). Slight differences in results were observed after normalization with housekeeping gene (Supplementary Figure S5). Protein levels of BGT were also examined, yet results were excluded from analysis due to poor BGT detection in skeletal and diaphragm tissues.

### 2.3. Localization of Osmolyte Pathway Members in Tibialis Anterior by Immunofluorescent Staining

**Figure 4:**
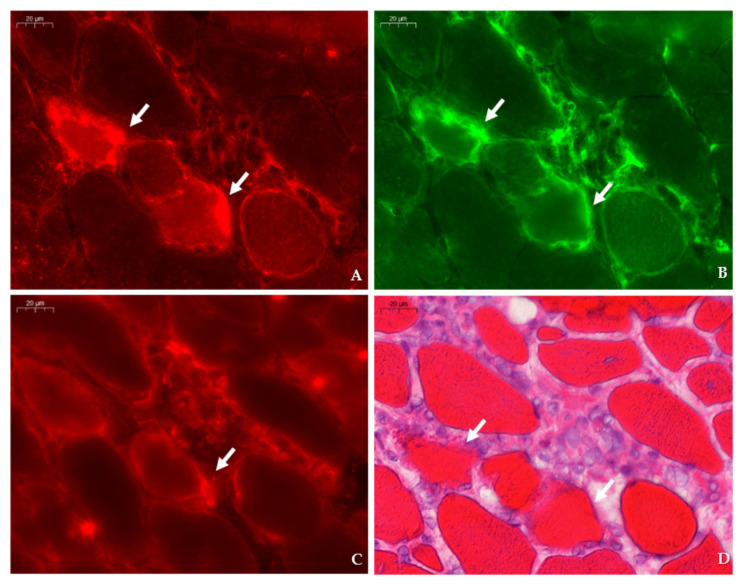
Expression of TauT in relation to dystrophinopathy-associated pathology in consecutive sections of the tibialis anterior in a 4 week-old *mdx* mouse. (**A**,**B**) Strong staining of TauT (CY3, red) is observed in myofibers (arrow) abutted by F4/F80 macrophages (AlexaFluor 488, green). (**C**) Staining of CD206 (CY3, red) in the consecutive section shows the presence of type II macrophages surrounding a myofiber (arrow) that corresponds to the TauT positive myofiber in panel A. (**D**) Identification of necrotic fibers (arrows) characterized by loss of membrane integrity stained with hematoxylin–eosin was performed in the same section as the immunofluorescent double stain (TauT/F4F80). Scale bars = 20 µm.

**Figure 5:**
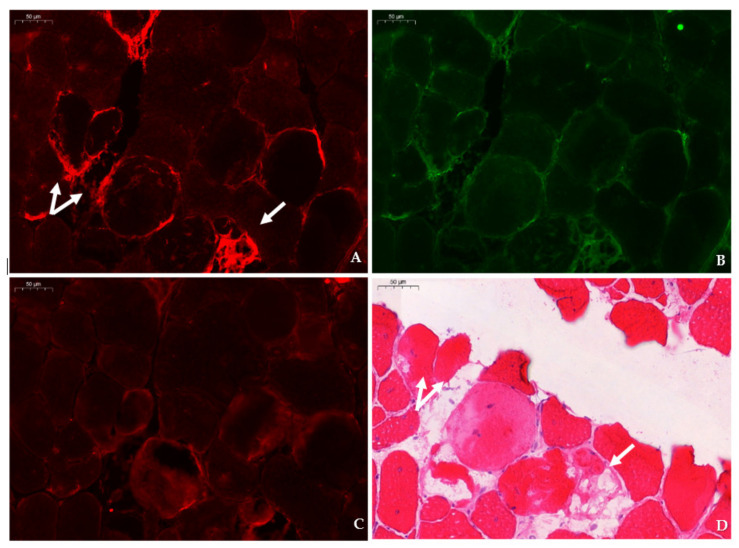
Expression of SMIT in relation to dystrophinopathy-associated pathology in consecutive sections of the tibialis anterior in a 12 week-old *mdx* mouse. (**A**) SMIT (CY3, red) is highly expressed in the membrane and the cytoplasm of a subset of myofibers (arrows). (**B**) Staining with F4/F80 antibody did not show the presence of F4/F80 macrophages situated in this field (AlexaFluor 488, green). (**C**) Staining with CD206 antibody (CY3, red) in the consecutive section was negative for type II macrophages. (**D**) Identification of fibers (arrows) in the same section that stained previously positive for SMIT in the double staining (SMIT/F4F80) show clear necrotic features in hematoxylin–eosin section. Scale bars = 50 µm.

**Figure 6:**
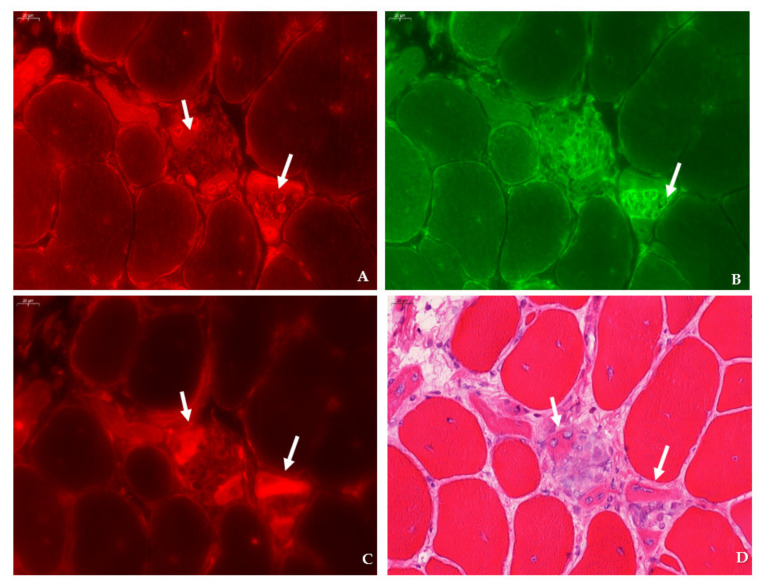
Expression of aldose reductase (AR) in relation to dystrophinopathy-associated pathology in consecutive sections of the tibialis anterior in a 12 week-old *mdx* mouse. (**A**) AR (CY3, red) is highly expressed in small regenerating myofibers (arrows). (**B**) F4/F80-positive macrophages (AlexaFluor 488, green) indicated by arrows, are located in the vicinity of AR positive myofibers. (**C**) Staining of a consecutive section with CD206 antibody (CY3, red, arrows) shows a clear colocalization with AR-positive fibers observed in panel A. (**D**) Identification of small regenerative fibers with central nuclei (arrows) in H&E section that stained previously positive for the presence of AR in the double stain (AR/F4F80). Scale bars = 20 µm.

**Figure 7:**
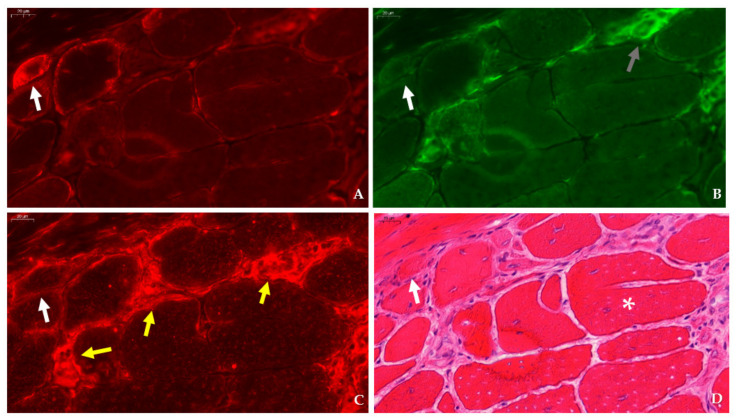
Expression of betaine GABA transporter (BGT) in relation to dystrophinopathy-associated pathology in consecutive sections of the tibialis anterior in a 12 week-old *mdx* mouse. (**A**) BGT (CY3, red) is expressed in a few myofibers (arrow). (**B**) F4/F80 positive macrophages (AlexaFluor 488, green, grey arrow) does not necessarily localize with BGT-positive myofibers (white arrow). (**C**) Staining of the consecutive section with CD206 antibody (CY3, red, yellow arrows) does not convincingly localize to the BGT-positive fiber (white arrow) observed in panel A. (**D**) After double staining with BGT/F4F80, this section was re-stained with H&E. The arrow indicates the BGT-positive fiber in panel A, whereas the asterisk shows a splitting fiber. Scale bars = 20 µm.

In dystrophic muscle, TauT was mainly localized to the sarcolemma, blood vessels, and on endomysial macrophages. Sarcoplasmic expression of TauT tended to be more prominent in myofibers challenged by surrounding and invading inflammatory F4/F80+ macrophages (Figure 4). Concurrently, induction of TauT in myofibers was observed in the vicinity of CD206+ macrophages and might be associated with the regenerative response to necrosis. Staining of SMIT (Figure 5) was observed at the sarcolemma of a subset of muscle fibers and showed no obvious colocalization with F4/F80+ or CD206+ macrophages. Some myofibers exhibited an intense but irregular staining pattern within their fragmented sarcoplasm. These myofibers showed necrotic characteristics, which were confirmed by staining with H&E. Within these myofibers, sporadic SMIT staining of invading and surrounding CD206 and F4/F80+ macrophages were observed. Similarly, discontinuous cytoplasmic expression of AR was observed in necrotic myofibers and was most prominent in small regenerating fibers in proximity of macrophages that showed F4/F80, and especially CD206 staining (Figure 6). Overall, weak continuous sarcolemmal and myonuclear expression of BGT was observed (Figure 7). In some myofibers, BGT expression coincided with the presence of CD206+ macrophages, and more rarely with F4/F80+ macrophages.

### 2.4. Gene Expression of Osmolyte Pathway Members in Tibialis Anterior of the Mdx Mouse Model at Different Time Points

No significant changes in mRNA expression of osmolyte pathway members in tibialis anterior could be observed between control and *mdx* mice, except for an upregulation of BGT in 8 week-old *mdx* mice. Increased mRNA expression of secreted phosphoprotein 1 (SPP1) was present in 4 and 8 week-old mice, whereas C-C motif chemokine ligand 2 (CCL2), also known as monocyte chemoattractant protein 1 (MCP-1), expression was upregulated in 12 week-old *mdx* mice (Supplementary Figure S6 and Table S1).

## 3. Discussion

Scientific evidence has accumulated for the involvement of osmotic stress in muscular dystrophy, and we previously observed upregulation of osmolyte pathway members TauT, SMIT, and AR in muscle tissue from DMD patients [6]. The aim of the current study was to investigate possible disease stage-related effects on osmolyte pathway activation in the *mdx* mouse model and, somewhat unexpectedly, our results revealed a differential regulation of individual osmolyte transporters, with upregulation of SMIT and downregulation of TauT protein levels in the *mdx* mouse model. These findings suggest a more complex regulation of osmolyte pathway members than general stress damage control in the *mdx* mouse model. Our results indicate variations of osmolyte pathway activation that seem to follow the trajectory of disease progression, with lower TauT protein levels in the most active phase of muscle degeneration/regeneration and normal levels in hind limb muscle of 26 week-old *mdx* mice, at which time muscle damage stabilizes. These results reveal complex and underappreciated involvements of osmolyte pathway members in DMD pathology, affirming the topic as a research interest and future therapeutic target in dystrophinopathy. This descriptive study in the *mdx* model contributes to the understanding of differences in pathological features between the mouse model and DMD patients.

Similar to published observations [13,14], we found necrotic hallmarks (3.6%) and myofibers with central nuclei (40.7%), indicative of regenerated fibers, in the tibialis anterior of 4 week-old *mdx* mice. At age 12 weeks, necrotic and regenerated fibers were observed in 9.2 and 59.9% of the myofibers, respectively. Our results are in line with previous studies that reported necrosis varying from 1 to 7% in 12 week-old *mdx* mice [15,16,17], whereas 70% of the myofibers exhibited a central nucleus at age 13 weeks [15]. In the adult *mdx* mouse, low graded necrosis has been described [7,18], which is consistent with our observations (≈3.1% of the myofibers affected) in 26 week-old *mdx* mice. Furthermore, the fraction of regenerated fibers increased to 64.2%. Higher levels of regenerative fibers might have been hypothesized; however, these fibers might be repeatedly subjected to necrosis [11], which could explain the non-linearity between the total amount of fibers subjected to necrosis and the percentage of regenerated fibers observed. These results indicate that necrosis was most prominent in skeletal muscle of 12 week-old *mdx* mice, and persists at slower pace in older mice. 

In muscle biopsies of young DMD patients, the amount of necrotic fibers remains stable at 1.5–1.8% in patients older than 3 years, and the percentage of fibers with centralized nuclei significantly increase from age 1 (1.31%) to 10 years (8.9%). Connective tissue (30%) is already highly abundant in young (7–10 years) DMD patients [19], a feature that becomes conspicuous in older *mdx* mice [7].

We were the first to recently show the involvement of NFAT-5-mediated pathways and general activation of its downstream osmolyte pathway members in the pathogenesis of DMD [6,20]. In the *mdx* mouse model, we observed that protein levels of TauT and SMIT were altered in *mdx* mice in comparison to age-matched controls, though without significant changes at the mRNA level. Presumably, these factors could be regulated via post-translation modifications [21,22].

TauT protein levels were significantly lower in the tibialis anterior and gastrocnemius of *mdx* mice aged 4, 8, and 12 weeks in comparison to age-matched control mice. These results were in line with the low levels of TauT reported in quadriceps of 18 and 42 day-old *mdx* mice [12]. In the skeletal muscles of older *mdx* mice (aged 26 weeks), TauT protein levels practically normalized to control levels, which coincided with lower graded necrosis. Protein levels of the transporter were significantly downregulated in the diaphragm of *mdx* mice as well, most explicitly at age 26 weeks. Unlike stabilization of dystrophinopathy in the skeletal muscle by age 26 weeks, damage to the diaphragm continuously increases with age [7], which could fit the tissue’s progressive decrease in TauT levels. Diminished levels of TauT, and subsequently a low intramuscular taurine concentration, are presumably associated with muscle wasting. In evidence, inhibition of TauT by guanodinoethane sulfonate supplementation in mice reduced taurine muscle content and resulted in a small but significant decline in force output when compared to controls [23]. Furthermore, *TauT knockout* mice suffer from muscle fatigability and increased serum creatine kinase levels [24], both hallmarks of disease in DMD. Our results indicate that protein levels of TauT are reduced, at which time degeneration/regeneration is most active and TauT could act as a biomarker. Given the detrimental effect of low TauT levels, diminished levels of taurine and/or its transporter might actively contribute to disease progression in the *mdx* mouse model; however, further investigation is required to support this theory.

Taurine supplementation appears to be effective in the *mdx* mouse model. *mdx* mice that received taurine treatment showed significantly higher numbers of intact myofibers with peripheral nuclei, together with reduced markers of inflammation and oxidative stress [25,26]. However, high doses of taurine have been shown to alter taurine synthesis in the liver, with unwanted growth restraint as a consequence [26]. Another possible limitation of taurine supplementation relates to the discrepancy in taurine regulatory pathways between the *mdx* mouse model and DMD patients. In contrast to our observations in the muscles of the *mdx* mouse, TauT has been described to be upregulated in muscle of 8 month-old *golden retriever muscular dystrophy* dogs [27] and DMD patients aged 4 to 9 years [6]. Presumably, the difference in TauT regulation is more likely species-specific than age-related, since the age of 3 to 4 week-old *mdx* mice corresponds to the age of 10 years in DMD patients [7]. Furthermore, patients with a homozygous mutation in the *SLC6A6* gene and subsequent low taurine levels suffer from retinal degradation, without any description of muscle impairment mentioned [28]. Additionally, chronic taurine supplementation in humans failed to increase muscle taurine content [29], compromising the possible benefit of long-term taurine treatment. These findings point towards strict regulation of endogenous osmolyte levels in the human body. Taurine function, metabolism, and compensatory mechanisms might vary between species. Up until now, taurine supplementation as a therapeutic strategy has not yet been tested in DMD patients. Both our former studies and others’ firmly link taurine metabolism to dystrophin deficiency. We suggest that the taurine pathway and its regulation in both *mdx* mice and DMD patients needs to be characterized further to successfully explore the therapeutic potential of taurine.

Upregulation of SMIT protein levels in the *mdx* mouse model is described here for the first time. In the skeletal muscles of *mdx* mice, SMIT protein levels were readily detected, whilst in control mice, the protein was almost undetectable, resulting in high fold changes. In the diaphragm, clear bands were present in both *mdx* and control mice, pointing to muscle subgroup-specific protein levels of SMIT. We noticed that the observed molecular weight (≈±53 kDa) was lower than the expected molecular weight of the full-length SMIT protein (≈79 kDa). This phenomenon might be explained by alternative splicing [30], post-translational modifications, or it can refer to the uncharacterized SMIT protein (≈58 kDa) known as (UniRef100_Q3UMR9) [31]. Immunofluorescent detection in the tibialis anterior of *mdx* mice revealed SMIT expression was most conspicuous in necrotic myofibers with fragmented sarcoplasm, whereas sporadic expression was observed in CD206+ and F4F80+ macrophages. Presumably, myofibers are the main source of SMIT expression. In DMD patients, upregulation of SMIT was observed at the protein level and colocalization studies showed that expression coincided with CD56+ fibers, a subset of CD206+ macrophages and T-cells, pointing towards involvement in the inflammatory and the successive regenerative response [6]. In vitro experiments have shown a significant upregulation of SLC5A3 mRNA in muscle cells exposed to pro-inflammatory cytokines [32]. Taken together, our results show increased protein levels of SMIT in *mdx* mice, especially in damaged muscle fibers. Supposedly, SMIT is upregulated in order to counteract osmotic imbalance, the latter being a consequence of leaky membranes that are conspicuous in dystrophin-deficient muscle [6]. Previously, upregulation of myo-inositol levels in *mdx* brain has been reported [33].

AR levels have been described to be upregulated in the skeletal muscle of DMD patients [6]. However, we could not detect significant differences in AR protein levels in skeletal muscle nor in the diaphragm of *mdx* mice, yet marked expression of AR was present in the necrotic myofibers and small, regenerating myofibers of *mdx* muscle tissues. Using liquid chromatography-mass spectrometry, a fold change of 1.38 in AR protein was reported in the heart of aging *mdx* mice when compared to 7 week-old *mdx* mice [34].

In conclusion, we revealed a substantial difference in regulation between TauT and SMIT, two members of the osmolyte pathway downstream of the NFAT-5 cascade, in the *mdx* mouse model. These results point towards a specific regulation of osmolyte pathways in the *mdx* mouse model, which are more complex than a general response to stress. In the *mdx* mouse model, active degeneration/regeneration occurs early in the species’ lifetime. Our results indicate that TauT protein levels during this period are diminished in the *mdx* mouse, whereas these levels normalize to control levels in older mice, at which time histopathological characteristics are less apparent. Whether TauT levels are affected by muscle damage or actively contribute to dystrophinopathology remains unclear. The results discussed in this paper highlight the potential of osmolyte pathway members as a research interest and future therapeutic target in dystrophinopathy.

## 4. Materials and Methods

### 4.1. Ethical Statement

Mice were bred at the central specific pathogen free animal facility of Ghent University (ECD 17/130). All experimental procedures were approved by the Animal Ethics Committee of Ghent University, faculty of Medicine and Health Sciences (ECD 19/77 and 19/110) and performed in accordance with European guidelines (Directive 2010/63/EU) and ARRIVE guidelines.

### 4.2. Animals

C57BL/10ScSn-Dmdmdx/J (*mdx*) mice and C57BL/10SnJ control mice were purchased from Jackson Laboratory (Bar Harbor, ME, US). All animals had access to food and water ad libitum. The male mice were selected, weighed, and euthanized by cervical dislocation at different ages: day 28(±1), 56(±1), 84 (±1), and 182 (±7) after birth. A total of 8 animals per age group (week 4, 8, 12, 26) of each strain were used for molecular experiments and 5 animals per age group of each strain for immunohistological analysis. Following sacrifice, specific skeletal muscle groups were dissected, those being the tibialis anterior, gastrocnemius, and diaphragm, and were then either isopentane (for histology) or snap (for other techniques) frozen, and stored at −80 °C until further processing. The number of siblings within age groups was kept as low as possible to avoid litter-dependent bias.

### 4.3. Western Blot

Tissue was ground in 2 units of extraction buffer (50 mM TrisHCL, 2mM EDTA pH 7.4) supplemented with protease and phosphatase inhibitor tablets (TM mini protease inhibitor cocktail and PhosStop; Roche, Indianapolis, IN, US). Muscle extracts were centrifuged twice at 15,000 RPM for 20 min, supernatant was collected, and protein concentration was measured using the Biodrop µLite device (Isogen, PW Utrecht, The Netherlands). Loading buffer, containing 2-mercapto-ethanol and 4x Laemmli sample buffer (Bio-Rad Laboratories, Hercules, CA, USA), was added to muscle extracts in a 1 to 3 ratio. Samples were boiled for 2 min and 15–20 µL of total lysate was loaded (Supplementary Table S2) onto 4–20% Mini-Protean TGX Stain-Free gel (Bio-Rad Laboratories, Hercules, CA, US) for electrophoresis. Next, proteins were transferred to ethanol-treated low fluorescent polyvinylidene membrane (Bio-Rad Laboratories, Hercules, CA, US). Prior to overnight antibody incubation, non-specific binding was avoided by 1 h treatment in a tris-buffered saline with 0.1% Tween20 (TBST) blocking solution containing 0.2% non-fat dry milk. The following antibodies were used: rabbit anti-GADPH antibody-loading control (0.2 µg/mL, ab 9485, Abcam, Cambridge, UK); rabbit anti-beta tubulin polyclonal antibody (0.5 µg/mL, PA5-16863, Invitrogen, Waltham, MA, US); rabbit anti-BGT-1 antibody (2 µg/mL, ab 200676, Abcam, Cambridge, UK); rabbit anti-SLC6A12 antibody (10 µg/mL, NBP1-88641, Novus Biologicals, Centennial, CO, US); rabbit anti-SLC6A12/BGT-1 antibody (10 µg/mL, LS-C411851, LifeSpan Biosciences, Seattle, WA, US); rabbit anti-AKR1B1 polyclonal antibody (1 µg/mL, PA5-12316, Invitrogen, Waltham, MA, US); rabbit anti- slc6a6/TauT antibody (0.125 µg/mL, ab 196821, Abcam, Cambridge, UK); rabbit anti-SLC5A3 antibody (2 µg/mL, ABS 518, Sigma-Aldrich, St. Louis, MO, US). Membranes were washed with TBST and exposed to the corresponding horseradish peroxidase-conjugated secondary antibodies. Protein bands were visualized with chemiluminescent Clarity Western ECL substrate (Bio-Rad Laboratories, Hercules, CA, US) or with the chromogenic Western Breeze kit (Invitrogen, Waltham, MA, US) using the Chemidoc Imaging System (Bio-Rad Laboratories, Hercules, CA, US), except for BGT, since no protein bands could be obtained by Western blotting using three different commercial antibodies. Densities were quantified with Image-Lab 6.0 software, normalized to total protein using Stain-Free technology (Bio-Rad Laboratories, Hercules, CA, US), or normalized against a housekeeping protein (GAPDH/beta tubulin) to correct for loading errors. Protein data per age category was analyzed relative to control animals (=1). Data of SMIT levels in the tibialis anterior, gastrocnemius, and diaphragm were log-transformed to adhere to normality criteria and univariate ANOVA analysis was performed. Other datasets were evaluated by mixed model analysis with the following specifications: the variables ‘group’, ‘age’, and ‘age by group’ were defined as fixed effects and were nested in this study. Bonferroni correction was used to compare relative differences in protein levels of osmolyte pathway in *mdx* and control mice of different ages.

### 4.4. qPCR

RNA was isolated from the tibialis anterior using the RNeasy Mini kit, following the manufacturer’s instructions (Qiagen, Hilden, Germany). In short, skeletal muscle tissue and Qiazol lysis reagent were added to tubes containing ceramic beads for homogenization with the Precellys system (Peqlab, Erlangen, Germany). Chloroform was added to muscle extracts prior to centrifugation. Next, supernatant was mixed with 200 µL of 70% EtOH solution and transferred to an RNEasy spin column. Washing steps were carried out according to the manufacturer’s protocol and each step was followed by centrifugation. RNAse free water was added to the spin column membrane, and, after centrifugation, purified RNA was obtained. RNA concentration was measured with a Nanodrop 1000 (ThermoFisher Scientific, Waltham, MA, US). Next, cDNA was prepared by adding 500 µg/mL oligo (dT)s, 5x First Strand Buffer, 0.1M DTT, 10mM dNTPs, and SuperScript II Reverse transcriptase (Invitrogen, Waltham, MA, US) according to the manufacturer’s protocol. PCR reactions were run in triplicates on a 7500 Real Time PCR system by supplementing 1 µL cDNA with 10 µL Taqman Gene Expression Mastermix (Applied Biosystems, Foster City, CA, USA), 8 µL RNAse free water, and 1 µL specific primers for: SLC5A3 (Mm00444330_s1), SLC6A6 (Mm00436909_m1), SLC6A12 (Mm00446665_m1), AKR1B3 (Mm01135578_g1), CCL2 (Mm00441242_m1), SPP1 (Mm00436767_m1), (Applied Biosystems, Waltham, MA, US). Analysis was carried out using Quantstudio and presented as 2^-DCT^ relative to GADPH mRNA expression. Results that displayed statistically significant changes in the first test were confirmed by a second round of qPCR reactions. Data were analyzed using the mixed model approach.

### 4.5. Histology

Left and right tibiales anteriores were carefully dissected and embedded in Tissue Tek O.C.T. (Sakura, Alphen aan den Rijn, The Netherlands) prior to snap freezing in isopentane-cooled nitrogen. Sections (8 µm) were cut transversally with a microtome and stained with H&E according to the protocol TREAT-NMD SOP: DMD_M.1.2.007. Whole muscle cross-sections were scanned and digitalized by the Pannoramic 250 Flash III (3DHistech, Budapest, Hungary) at a 20× enlargement. Sections were manually analyzed for the following parameters: number of healthy muscle fibers, number of regenerated muscle fibers with centralized nuclei, and number of necrotic myofibers (fibers invaded by macrophages, associated with loss of membrane integrity, and/or pale cytoplasm). In addition, non-muscle area (including necrotic surface, inflammatory regions, and interstitial area) was quantified both manually and automatically using color deconvolution with QuPath 0.2.3 software [35]. In total, 5 sections per animal (*n* = 5 animals per age group) with an interval of approximately 150 µm between consecutive sections were cut, stained, and analyzed. Per section, an average of 2338 fibers, representing a surface of 4.67 × 10^6^ µm^2^, were examined. The number of healthy, regenerating, and necrotic fibers to total fibers was compared between *mdx* and control groups of a specified age using the negative binomial regression model with log link distribution and compound symmetry as covariance structure. The variable ‘section’ was defined in repeated measures, since 5 sections were analyzed per animal. Log total fibers was used as offset to correct for the total amount of fibers counted per section. Fixed effects were determined by ‘group’, ‘age’, and ‘age by group’ and Bonferroni correction was used to compare results between different ages.

### 4.6. Immunofluorescence

Sections were permeabilized for 2 min in acetone and left to dry. Next, sections were incubated with PBS blocking solution that contained 2% bovine serum, 5% donkey serum, and 10% heat-inactivated human serum. The following primary antibodies were diluted in PBS blocking serum and incubated overnight at 4 °C: rabbit anti-SLC5A3 antibody (20 µg/mL, ABS 518, Sigma-Aldrich, St. Louis, MO, US), rat anti-F4/F80 antibody (5 µg/mL, ab6640, Abcam, Cambridge, UK), rabbit anti-CD206 antibody (400 µg/mL, PA5-101657, Invitrogen, Waltham, MA, US), rabbit anti-SLC6A12 antibody (400 µg/mL, NBP 1-88641, R&D systems, Minneapolis, MN, US) rabbit anti- SLC6A6/TauT (20 µg/mL, LS-C386313, LifeSpan Biosciences, Seattle, WA, US) and rabbit anti-AKR1B1 polyclonal antibody (400 µg/mL, PA5-12316, Invitrogen, Waltham, MA, US), goat anti-NCAM/CD56+ antibody (5 µg/mL, AF 2408, R&D systems, Minneapolis, MN, US), or rabbit anti-MYH8 (20 µg/mL, PA5-72846, ThermoFisher Scientific, Waltham, MA, US). Sections were consecutively stained with rat anti-F4/F80 in combination with rabbit antibody, directed to an osmolyte pathway member, and rabbit anti- CD206 antibody in combination with goat anti-NCAM/CD56 antibody. Sections were rinsed three times with PBS and incubated for 1 h with secondary antibodies labelled with CY3 (Jackson ImmunoResearch Laboratories, Westgrove, PA, US) and AlexaFluor 488 (ThermoFisher Scientific, Waltham, MA, US). Sections were rinsed three times with PBS and mounted with Fluoromount G (Southern Biotech, Birmingham, AL, USA). After digitalization, sections double stained for osmofactors and F4/F80 were subsequently stained with hematoxylin–eosin.

### 4.7. Statistical Analysis

Statistical analysis was performed in IBM SPSS Statistics version 27.0.0. Normality assessment was based on raw data and residual data. Statistical significance was set at a *p*-value of ≤ 0.05.

## Figures and Tables

**Table 1:** SMIT fold changes in skeletal muscles of mdx mice relative to controls.

Age	Tibialis Anterior	Gastro-Cnemius	Diaphragm
W04	453.9	7.7	2.1
W08	13.4	436.5	2.0
W12	9.85	71.0	3.2
W26	11.5	4.7	1.3

## Data Availability

Not applicable.

## Supplementary Materials

The following supporting information can be downloaded at: https://www.mdpi.com/article/10.3390/ijms23063251/s1.
