# Supplementary material for: Description of Osmolyte Pathways in Maturing Mdx Mice Reveals Altered Levels of Taurine and Sodium/Myo-Inositol Co-Transporters
Source: Int J Mol Sci. 2022 Mar 17;23(6):3251. doi: 10.3390/ijms23063251 (PMC8955384; doi:10.3390/ijms23063251)
Supplement: Supplementary file 1 [file ijms-23-03251-s001.zip › ijms-1629365-supplementary.pdf]

Supplementary files

Figure S1. Assessment of non-muscle area in tibialis anterior of *mdx* and control mice on age 4, 8, 12 and 26 weeks.

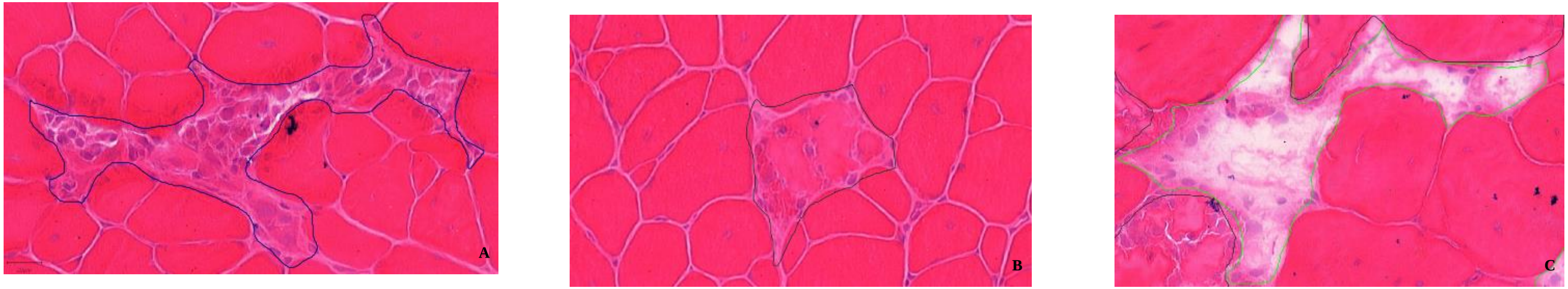

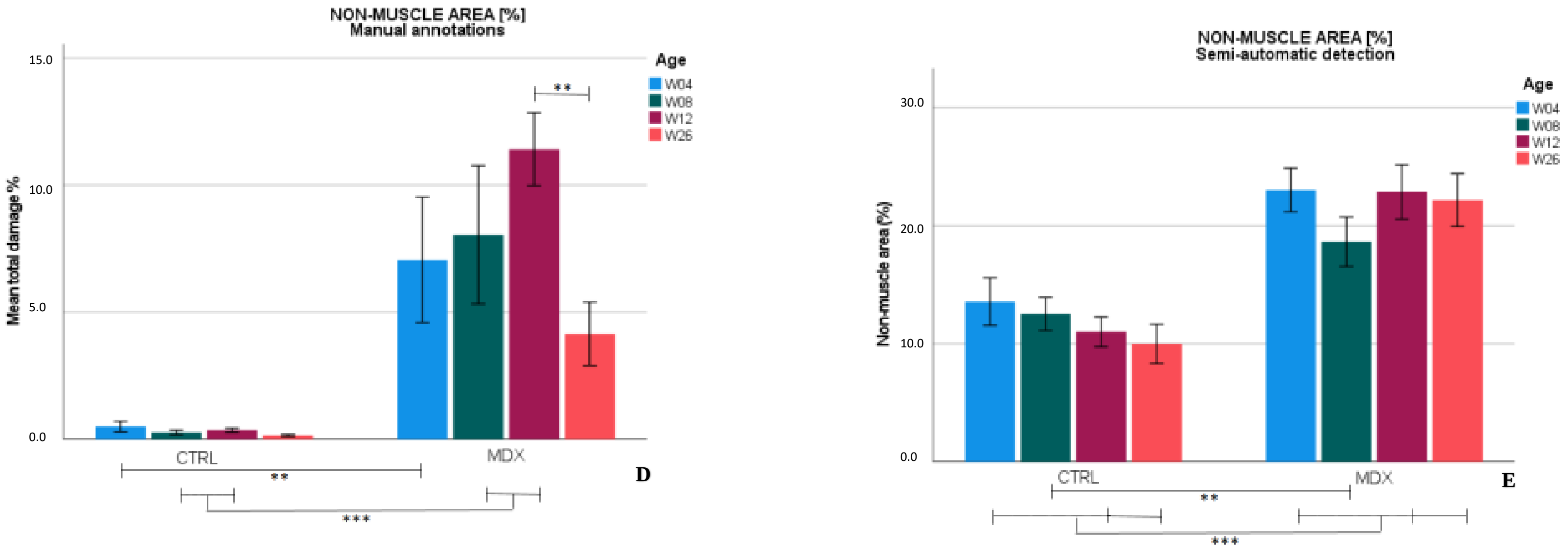

Non-muscle area was defined by (**a**) infiltrate of immune cells (blue), (**b**) necrotic cells (grey), and (**c**) interstitial area with pathological characteristics (green) in the manual annotated version.**(d)** Representation of mean % non-muscle area (including, necrotic surface, infiltrate area and interstitial space with pathological characteristics) after manual annotation in QuPath. **(e)** Percentage of non-muscle area according obtained after the colour deconvolution method in QuPath. In total 199 whole muscle sections were analysed. Data is shown as the mean percentage of non-muscle area ± standard error of the mean and n = 7-8. Significant differences are annotated by * ($p \leq 0.05$), ** ($p \leq 0.01$) and ***($p \leq 0.001$).

Figure S2. Relative protein levels of the taurine transporter (TauT) in skeletal muscle and diaphragm of *mdx* and control mice aged 4, 8, 12, and 26 weeks after normalization against glyceraldehyde 3-phosphate dehydrogenase (GAPDH).

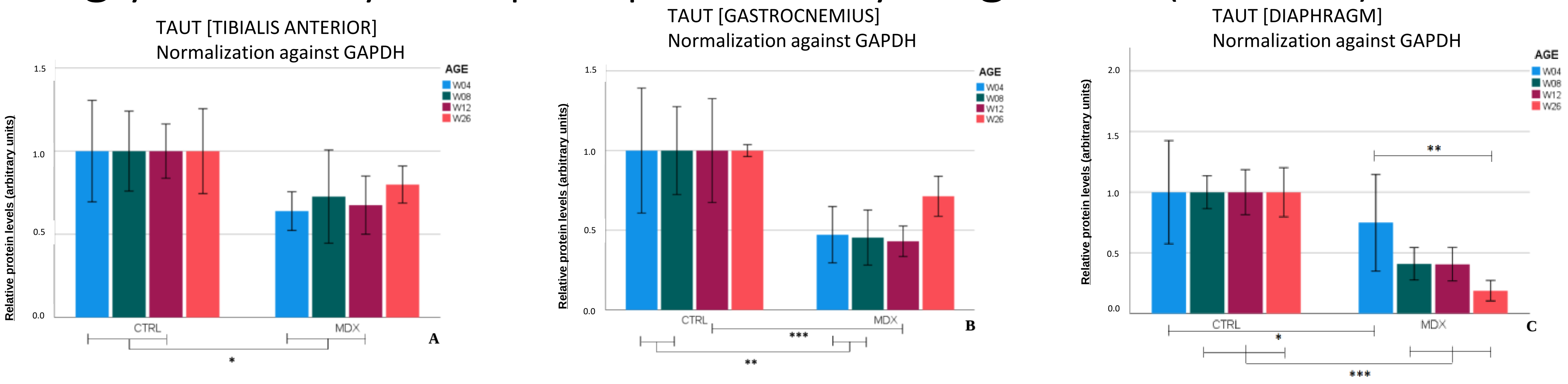

(a) Relative protein levels of TauT in tibialis anterior. **(b)** Relative protein levels of TauT in gastrocnemius. **(c)** Relative protein levels of TauT in diaphragm (n=7-8). Data was normalized against the housekeeping protein GAPDH. Data is shown as mean ± standard error of the mean (SEM) and n = 7-8. Significant differences are annotated by * ($p \leq 0.05$), ** ($p \leq 0.01$) and ***($p \leq 0.001$).

Figure S3. Relative protein levels of Sodium/myo-inositol transporter (SMIT) in skeletal muscle and diaphragm of *mdx* and control mice aged 4, 8, 12, and 26 weeks after normalization against GAPDH.

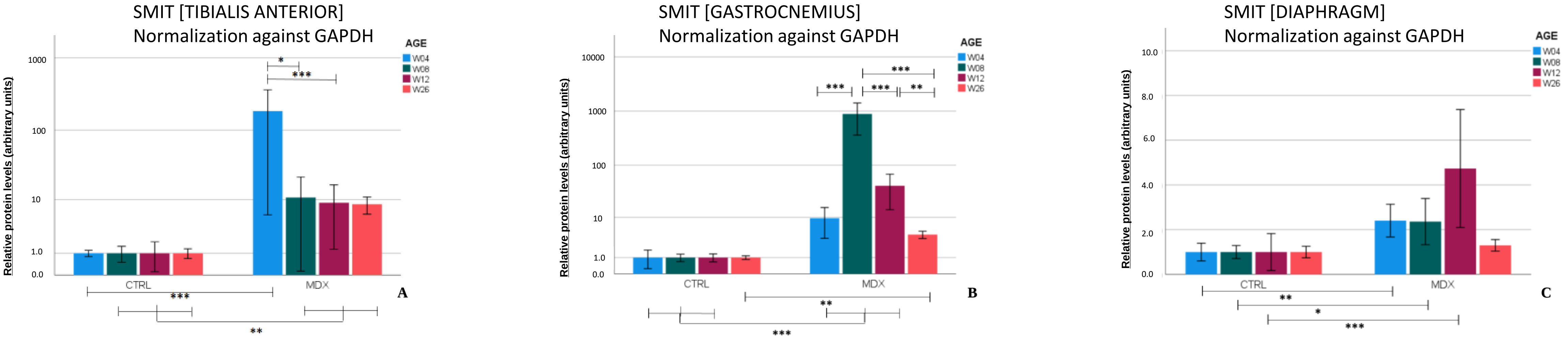

(a) Relative protein levels of SMIT in tibialis anterior. **(b)** Relative protein levels of SMIT in gastrocnemius. **(c)** Relative protein levels of SMIT in diaphragm (n=7-8). Data was normalized against the housekeeping protein GAPDH. Data is shown as mean ± standard error of the mean (SEM). Significant differences are annotated by * ($p \leq 0.05$), ** ($p \leq 0.01$) and ***($p \leq 0.001$).

Figure S4. Relative protein levels of aldose reductase (AR) in skeletal muscle and diaphragm of *mdx* and control mice.

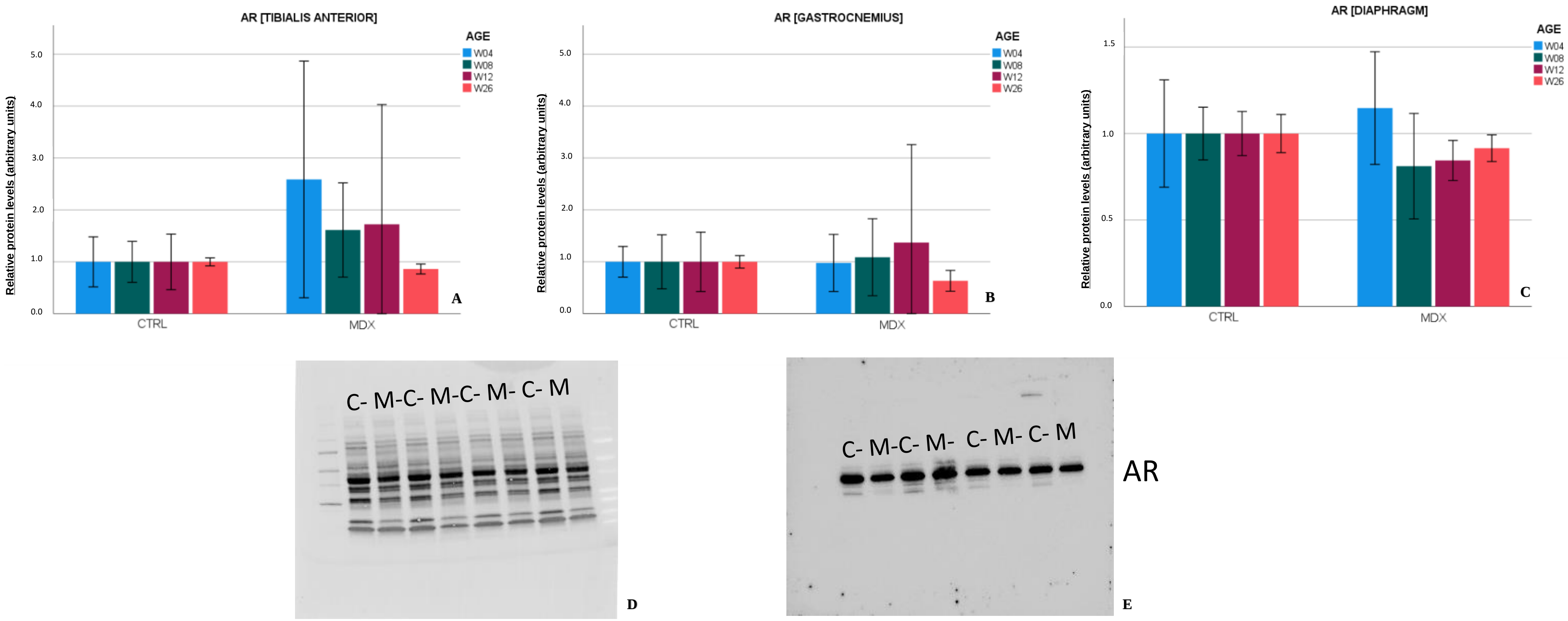

Protein levels of AR in mdx mice were reported relative to protein levels in control mice within the same age category. **(a)** Relative protein levels of SMIT in tibialis anterior. **(b)** Relative protein levels of SMIT in gastrocnemius. **(c)** Relative protein levels of SMIT in diaphragm (n=8). Data is shown as mean ± standard error of the mean (SEM). Significant differences are annotated by * ($p \leq 0.05$), ** ($p \leq 0.01$) and ***($p \leq 0.001$). Representative image of total protein detected in the diaphragm of 12-week-old mdx and control mice by Stain-Free technology that was used to correct for variation in loading **(d)** and the corresponding image of AR protein imaged by chemiluminescent detection **(e).**

Figure S5. Relative protein levels of aldose reductase in skeletal muscle and diaphragm of *mdx* and control mice normalized against B-tubulin.

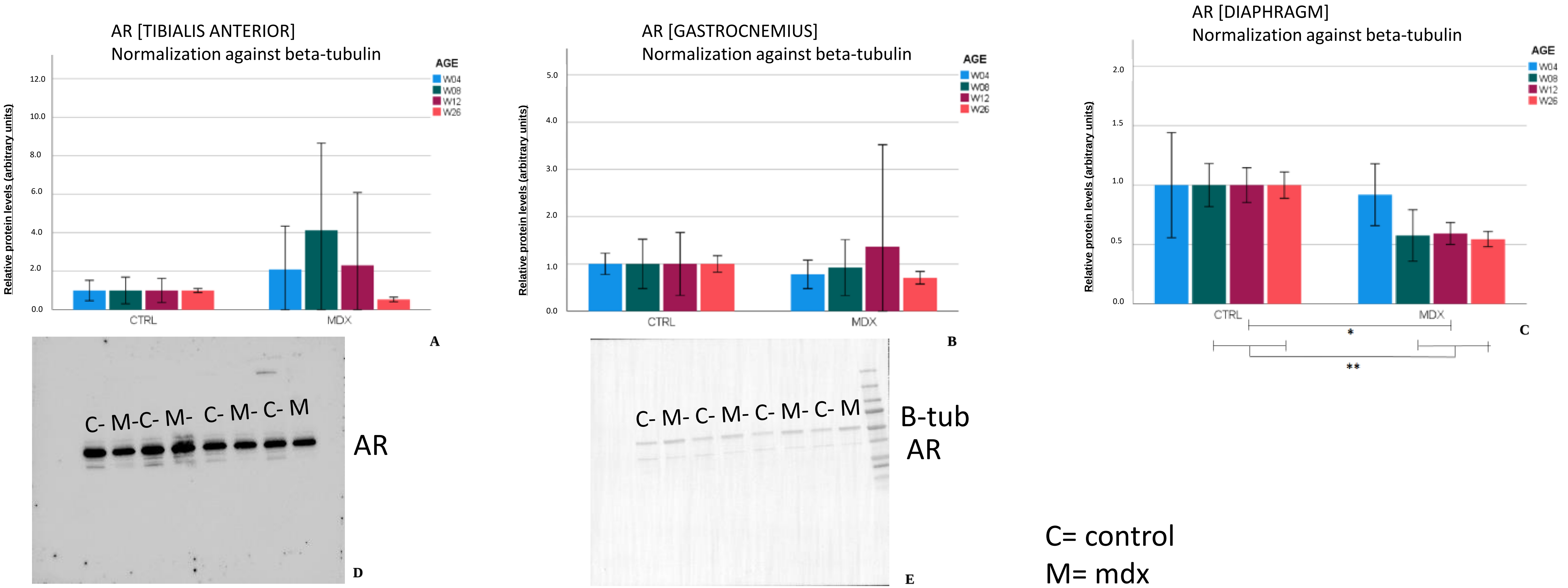

Protein levels of AR in *mdx* mice were reported relative to expression in control mice within the same age category.
(a) Relative protein levels of SMIT in tibialis anterior. **(b)** Relative protein levels of SMIT in gastrocnemius. **(c)** Relative protein levels of SMIT in diaphragm. Data was normalized against the housekeeping protein B-tubulin. Data is shown as mean ± standard error of the mean (SEM) and n = 7-8. Significant differences are annotated by * ($p \leq 0.05$), ** ($p \leq 0.01$) and ***($p \leq 0.001$). Representative image of AR protein in the diaphragm of 12-week old mice imaged by chemiluminescent detection **(d)** and B-tubulin imaged by colorimetric detection, which was used for normalization**(e).**

Figure S6. Gene expression in tibialis anterior of *mdx* and control mice at age 4, 8, and 12. Run 1

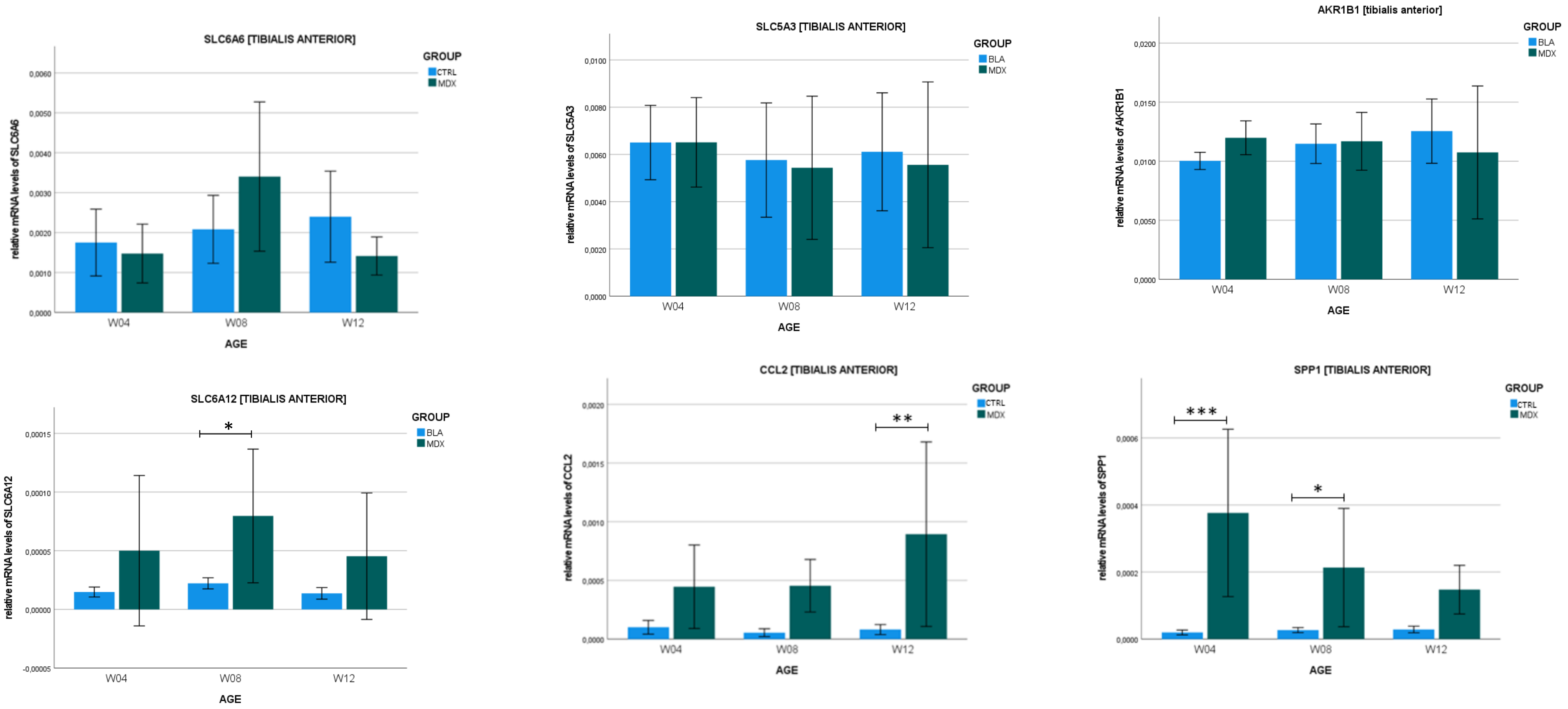

Analysis was carried out using Quantstudio, and data was presented as 2-ΔCT mean ± standard error of the mean (SEM) relative to GADPH mRNA expression. Significant differences are annotated by * ($p \leq 0.05$), ** ($p \leq 0.01$) and ***($p \leq 0.001$).

Table S1 : Overview of qPCR data in tibialis anterior of 4-, 8-, and 12- week old control and *mdx* mice.

	AKR1B1	SLC5A3	SLC6A6	SLC6A12	CCL2	SPP1
Mean value	CTRL W4: 0.010<br>MDX W4: 0.012<br><br>CTRL W8 0.011<br>MDX W8 0.012<br><br>CTRL W12 0.013<br>MDX W12 0.011	CTRL 4W 0.007<br>MDX 4W 0.007<br><br>CTRL 8W 0.006<br>MDX 8W 0.005<br><br>CTRL 12W 0.006<br>MDX 12W 0.006	CTRL 4W 0.002<br>MDX 4W 0.001<br><br>CTRL 8W 0.002<br>MDX 8W 0.003<br><br>CTRL 12W 0.002<br>MDX 12W 0.001	CTRL 4W 0.00001<br>MDX 4W 0.00005<br><br>CTRL 8W 0.00002<br>MDX 8W 0.00008<br><br>CTRL 12W 0.00001<br>MDX 12W 0.00005	CTRL 4W 0.0001<br>MDX 4W 0.0004<br><br>CTRL 8W 0.00005<br>MDX 8W 0.00046<br><br>CTRL 12W 0.00008<br>MDX 12W 0.00100	CTRL 4W 0.00002<br>MDX 4W 0.00030<br><br>CTRL 8W 0.000027<br>MDX 8W 0.000213<br><br>CTRL 12W 0.000030<br>MDX 12W 0.000148
P-value	W4 0.345<br>W8 0.921<br>W12 0.384	W4 0.996<br>W8 0.858<br>W12 0.763	W4 0.721<br>W8 0.091<br>W12 0.204	W4 0.236<br>**W8 0.041**<br>W12 0.250	W4 0.189<br>W8 0.129<br>**W12 0.003**	**W4 <0.001**<br>**W8 0.046**<br>W12 0.198

A

	AKR1B1	SLC6A6	SLC6A12	CCL2	SPP1
Mean value	CTRL W4: 0.02<br>MDX W4: 0.01	CTRL W12 0.002<br>MDX W12 0.002	CTRL 8W 0.00001<br>MDX 8W 0.00007	CTRL W8 0.00006<br>MDX W8 0.00041<br><br>CTRL W12 0.00007<br>MDX W12 0.00075	CTRL W4: 0.00003<br>MDX W4: 0.00025<br><br>CTRL W8 0.00004<br>MDX W8 0.00026<br><br>CTRL W12 0.00003<br>MDX W12 0.00015
P-value	W4 0.384	W12 0.462	**W8 0.039**	W8 0.072<br>**W12 0.001**	**W4 0.034**<br>**W8 0.035**<br>W12 <0.001

B

(a) Results qPCR run 1; performed in triplicates.

(b) Results qPCR confirmational run 2; performed in triplicates. Results that are underlined show significant results in at least 1 run. Results that are in bold and underlines show significant results in both runs.

Table S2 : Overview of protein loading.

		Protein concentration (µg/ml)	Loading (µl)	Loading (µg)
Tibials anterior	Week 4	853,3	20	17
	Week 8	807,8	20	16
	Week 12	925,4	20	18,5
	Week 26	1000	15	15
Gastrocnemius	Week 4	1654,5	15	25
	Week 8	2236,9	15	33,5
	Week 12	2136,9	15	32
	Week 26	1000	15	15
Diaphragm	Week 4	1000	15	15
	Week 8	1000	15	15
	Week 12	1000	15	15
	Week 26	1000	15	15

Table S2: overview of protein loading per gel. Protein concentrations (µg/ml) and loading (µl; µg) are provided in this table. Of note, within one gel the same protein concentration and loading was maintained. Normalization was carried out with stainfree blot technology to correct for variation in loading and data was reported in reference to control values which made comparison between age/tissue possible.
